# Supplementary material for: Cryopreservation of lumpfish Cyclopterus lumpus (Linnaeus, 1758) milt
Source: PeerJ. 2015 Jun 4;3:e1003. doi: 10.7717/peerj.1003 (PMC4458125; doi:10.7717/peerj.1003)
Supplement: Table S1 — Three different cryosolutions were tested in the pilot study. The chemical composition of the diluents and cryoprotectants are shown in the table along with the dilution factor (milt:diluent) used. The volume of every cryoprotectant was determined based on the volume of the diluents loaded. [file peerj-03-1003-s001.docx]

| Cryos.: | Source: | Diluents: | Dilution: | Cryoprotectant: |
| --- | --- | --- | --- | --- |
| 1 | (Rideout, Trippel & Litvak, 2004) | 0.137 M NaCl, 0.011 M KCl, 0.004 M (Na_2_HPO_4_)7H_2_O | 1:3 | 10% Propylene glycol |
| 2 | (Ding et al., 2011) | 0.1 M KHCO_3_ and 0.125 M sucrose | 1:3 | 10% DMSO |
| 3 | (Zhang et al., 2003) | 423 mM NaCl, 8.99 mM KCl, 9.25 µM CaCl_2_.2H_2_O, 22.92 µM MgCl_2_-6H_2_O, 25.52 µM MgSO_4_.7H_2_O, and 2.14 µM NaHCO_3_ | 1:2 | 12% Glycerol |

All reagents were purchased from VWR, Bie & Berntsen, Denmark.
