# Supplementary material for: Cryopreservation of lumpfish Cyclopterus lumpus (Linnaeus, 1758) milt
Source: PeerJ. 2015 Jun 4;3:e1003. doi: 10.7717/peerj.1003 (PMC4458125; doi:10.7717/peerj.1003)
Supplement: Table S2 — Milt cryopreserved in three different cryosolutions were tested with the first freezing and thawing procedure (trey height: 2.5 cm; thawed at 50 °C for 1 min). Only spermatozoa cryopreserved in cryosolution 2 did have any motility recovery, and therefore only spermatozoa cryopreserved in this solution were tested with the second freezing and thawing procedure (trey height: 4.5 cm; thawed at 37 °C for 1.5 min). Motility of fresh milt was measured before the freezing and thawing procedures and was evaluated a motility score of 4 (moving cells: 76–100%). [file peerj-03-1003-s002.docx]

| **First freezing and thawing procedure** | | |
| --- | --- | --- |
| Cryosolutions: | Motility score: | Moving cells: |
| 1 | 0 | 0 |
| 2 | 1 | 1-25% |
| 3 | 0 | 0 |
|  |  |  |
| **Second freezing and thawing procedure** | | |
| Cryosolutions: | Motility score: | Moving cells: |
| 2 | 3 | 50-75% |
