# Supplementary material for: Cryopreservation of lumpfish Cyclopterus lumpus (Linnaeus, 1758) milt
Source: PeerJ. 2015 Jun 4;3:e1003. doi: 10.7717/peerj.1003 (PMC4458125; doi:10.7717/peerj.1003)
Supplement: Table S3 — Motility percentage of fresh milt and volume in ml (Vol.) obtained after each stripping (Batch) from individual lumpfish (Male) used in this study either captured wild (W) or from own brood stocks (B). [file peerj-03-1003-s003.docx]

| **Male** | **Batch** | **origin** | **vol.** | **Motility** |
| --- | --- | --- | --- | --- |
| **1** | a | W | 1.2 | 85.0 |
| **2** | b | B | 2.0 | 98.0 |
|  | c |  | 1.0 | 80.0 |
| **3** | d | B | 0.3 | 73.0 |
|  | e |  | 2.7 | 78.0 |
|  | f |  | 1.1 | 78.0 |
| **4** | g | B | no data | 59.0 |
| **5** | h | B | 0.3 | 87.0 |
|  | i |  | 0.5 | 71.0 |
|  | j |  | 0.5 | 65.0 |
| **6** | k | B | 0.6 | 83.0 |
|  | l |  | 0.6 | 66.5 |
|  | m |  | 0.6 | 69.0 |
| **7** | n | W | 2.0 | 76.0 |
|  | o |  | 0.9 | 42.7 |
|  | p |  | 0.3 | 54.0 |
