# Supplementary material for: Cryopreservation of lumpfish Cyclopterus lumpus (Linnaeus, 1758) milt
Source: PeerJ. 2015 Jun 4;3:e1003. doi: 10.7717/peerj.1003 (PMC4458125; doi:10.7717/peerj.1003)
Supplement: Table S4 — Male batch (milt), female batch (roe), motility (mot. %) and fertilization (fert. %) data of roe fertilized with either fresh (F) or cryopreserved (C) milt. Cryopreserved milt in this table is treated with Mounib no GSH using trey height 4.8 cm. The average motility recovery (mean ± SD) of fresh milt is 67.20 ± 16.16 and 53.45 ± 18.55 for cryopreserved milt. The average fertilization success using fresh milt is 30.81 ± 16.87 and using cryopreserved milt 27.30 ± 17.02. Fertilization success using fresh milt is not significantly higher than using cryopreserved milt (F1,24 = 0.91, P = 0.35). [file peerj-03-1003-s004.docx]

| **Milt** | **Roe** | **F/C** | **Mot. (%)** | **Fert. (%)** |
| --- | --- | --- | --- | --- |
| d | 30 | F | 73 | 48 |
| e | 29 | F | 78 | 28 |
| f | 31 | F | 78 | 0.0 |
|  | 35 |  |  | 60.7 |
|  | 28 |  |  | 46 |
|  | 23 |  |  | 8.2 |
| h | 33 | F | 87 | 41.1 |
|  | 34 |  |  | 16.7 |
| j | 40 | F | 65 | 45 |
| r | 39 | F | 47 | 41 |
| k | 33 | F | 83 | 27.2 |
|  | 34 |  |  | 21.5 |
| m | 40 | F | 69 | 19 |
| p | 40 | F | 54 | 41 |
| s | 39 | F | 38 | 20 |
| B | 30 | C | 37 | 47 |
| T | 30 | C | 92 | 50 |
| E | 29 | C | 83 | 42 |
|  | 31 |  |  | 0.0 |
|  | 35 |  |  | 14 |
|  | 28 |  |  | 3.2 |
|  | 23 |  |  | 3.5 |
| H | 33 | C | 58 | 62.9 |
|  | 34 |  |  | 40.3 |
| J | 40 | C | 48 | 31 |
|  | 39 |  |  | 25 |
| K | 33 | C | 39 | 29.6 |
|  | 34 |  |  | 19 |
| M | 40 | C | 55 | 41 |
|  | 39 |  |  | 17 |
| O | 40 | C | 42 | 13 |
| P | 40 | C | 34 | 29 |
| S | 39 | C | 47 | 25 |
| W | 39 | C | 53 | 25 |
