# Supplementary figures and images for: Cryopreservation of lumpfish Cyclopterus lumpus (Linnaeus, 1758) milt
Source: PeerJ. 2015 Jun 4;3:e1003. doi: 10.7717/peerj.1003 (PMC4458125; doi:10.7717/peerj.1003)

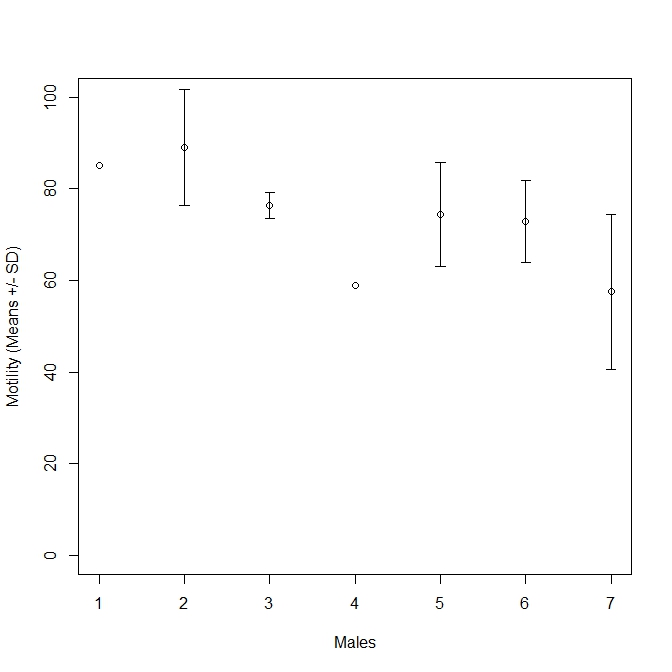

Supplement: Figure S1 — Motility of fresh milt split by males given as mean motility % ± SD. [file peerj-03-1003-s005.jpeg]
