# Supplementary material for: Cryopreservation of lumpfish Cyclopterus lumpus (Linnaeus, 1758) milt
Source: PeerJ. 2015 Jun 4;3:e1003. doi: 10.7717/peerj.1003 (PMC4458125; doi:10.7717/peerj.1003)
Supplement: Supplemental Information 6 — This is a short write-up of the methods used in our pilot study. Some references are given, that are perhaps not included in the main manuscript, so there is a reference list included in the pilot study methods. [file peerj-03-1003-s006.docx]

Pilot study methods

Two replicates of each cryosolution (supplementary table 1) were prepared in 2 ml cryogenic vials. The vials were first loaded with the diluents and milt dilution, after that the cryoprotectant was added. All milt samples were stripped and obtained from one male (2012). Fresh milt was used as a control.

Two procedures were tested in order to obtain an optimal freezing and thawing method. The freezing process was carried out in a styrofoam box (inside dimensions H x L x W= 21cm x 35.5cm x 23cm) with liquid nitrogen (-196º C) filled up to about 10 cm. During the freezing procedures, the cryogenic vials were placed upright on styrofoam trays at different heights using a metal wire attached to each vial and the tray. The tray and vials were placed on the liquid nitrogen in the styrofoam container in nitrogen gas for 10 minutes. They were then plunged directly into the liquid as the tray was turned over with a 20 cm long forceps. The vials were suspended in this position for at least 15 minutes. In the first freezing procedure, the height of the styrofoam tray was 2.5 cm, and, in the second freezing procedure, the height was changed to 4.5 cm. Subsequently the vials were thawed in water baths. The thawing temperature in the first procedure was 50^˚^ C and the duration time 1 minute. In the second procedure, the thawing temperature was reduced to 37^o^C and the duration time increased to 1.5 minute.

The motility score of milt samples was measured after the thawing procedure. We divided the movement of spermatozoa into five stages and classified each stage according to an arbitrary scale commonly used from 0 to 4 based on the percentage of moving cells (Viveiros, Jatzkowski & Komen, 2003; Rurangwa et al., 2004; Kainin et al., 2012) (Supplementary table 2).

References:

Ding F, Lall SP, Li J, Lei J, Rommens M, Milley JE. 2011. Cryopreservation of sperm from Atlantic halibut (Hippoglossus hippoglossus, L.) for commercial application. *Cryobiology*.

Kainin S, Ponchunchoovong S, Imsilp U, Singsee S. 2012. Cryopreservation of Mekong catfish, Pangasius bocourti Sauvage, 1880 spermatozoa. *Aquaculture Research*.

Rideout RM, Trippel EA, Litvak MK. 2004. The development of haddock and Atlantic cod sperm cryopreservation techniques and the effect of sperm age on cryopreservation success. *Journal of fish biology* 65:299–311.

Rurangwa E, Kime DE, Ollevier F, Nash JP. 2004. The measurement of sperm motility and factors affecting sperm quality in cultured fish. *Aquaculture* 234:1–28.

Viveiros ATM, Jatzkowski A, Komen J. 2003. Effects of oxytocin on semen release response in African catfish (Clarias gariepinus). *Theriogenology* 59:1905–1917.

Zhang YZ, Zhang SC, Liu XZ, Xu YY, Wang CL, Sawant MS, Li J, Chen SL. 2003. Cryopreservation of flounder (Paralichthys olivaceus) sperm with a practical methodology. *Theriogenology* 60:989–996.
